# Navigating new normals: the influence of COVID-19 policies on community access and well-being of people with mobility disabilities in everyday life

**DOI:** 10.3389/fpubh.2024.1401777

**Published:** 2024-07-04

**Authors:** Carrie L. Wendel, Randi Christine Gray, Kelsey Goddard, Jean P. Hall

**Affiliations:** ^1^School of Social Welfare, University of Kansas, Lawrence, KS, United States; ^2^Department of Educational Psychology, Counseling Psychology, University of Kansas, Lawrence, KS, United States; ^3^Research and Training Center on Independent Living, Institute for Health and Disability Policy Studies, University of Kansas, Lawrence, KS, United States

**Keywords:** disability, COVID-19, pandemic, policy, qualitative, public health adherence

## Abstract

**Context:**

This study explores the influence of COVID-19 public health mandates on people with mobility disabilities in the United States in their everyday lives. It highlights the intersection of disability with social determinants of health, emphasizing the need for a comprehensive policy response.

**Methods:**

Qualitative data were collected through 76 semi-structured interviews with people with mobility disabilities. Interviews focused on experiences with COVID-19 mandates and community access, analyzed using thematic analysis and coded for emergent subthemes.

**Results:**

The relationship between community participation and COVID-19 compliance was complex for people with disabilities. Inaccessible environments and inflexible policies made it difficult for people with disabilities to practice good safety measures, while widespread noncompliance by community members limited their community participation. The findings revealed additional mixed lived experiences of COVID-19 policies on community participation, accessibility, and access to resources and support. While technology facilitated some aspects of community participation, issues with accessibility, public transportation, and personal assistance services were exacerbated.

**Conclusion:**

COVID-19 policies have complex implications for people with mobility disabilities. Findings suggest a need for inclusive policymaking, improved disability awareness, and continued support for accessible technology and services. Future research should further explore these dynamics to inform policy and practice.

## Introduction

In response to the COVID-19 pandemic, public health mandates and safety guidelines were implemented to slow disease spread and save lives. These were particularly crucial for protecting high-risk groups such as older adults and individuals with preexisting health conditions or disabilities. While there is a growing body of literature exploring the impact of these mandates on people with intellectual and developmental disabilities (IDD), the specific effects on the daily lives and community engagement of people with disabilities (PWD), especially those with mobility disabilities, remain less explored and understood (1). Moreover, the increased risk of COVID-19 for PWD was not just due to preexisting health conditions, but also linked to social determinants of health like discrimination, healthcare access, economic resources, transportation, and other supports (2–4).

The United States National Institutes of Health recently designated people with disabilities as a health disparity group due to the combination of health conditions and social inequities that lead to poorer health outcomes for this population (5). Systemic barriers impacting the health, economic security, and social engagement persist for PWD, despite legislation such as the Americans with Disabilities Act (ADA). These include limited accessibility in public infrastructure, challenges in accessing healthcare due to high costs and physical and attitudinal barriers, and socio-economic obstacles that restrict their participation in society. Mental health issues are prevalent among PWD, stemming from social isolation and the psychological impacts of non-inclusivity (6). The states of Georgia, Indiana, Ohio, and Pennsylvania, where this study’s interviews were conducted, exhibit these national issues, with urban areas typically providing better resources than rural ones. The COVID-19 pandemic has exacerbated these disparities, highlighting the urgent need for comprehensive supports and inclusive policies to address the unique challenges for PWD during public health crises (7).

The mental health impact of social isolation from social distancing and stay-at home mandates has been widely observed (8, 9), including among PWD (1, 10, 11). These mandates also affected access to healthcare and essential services, with households having a disabled member more likely to report challenges in accessing basic needs during the pandemic such as food, housing, healthcare, transportation, medication, and stable income (12). Survey participants in a study by Goddard et al. (10) noted reduced access to healthcare and mental health care services, medications, and medical supplies. Inflexible policies, such as telehealth limits, inflexible retail policies, office closures, and staffing reductions, created avoidable barriers for PWD in accessing necessary care. Concerns about the virus and efforts to minimize exposure also reduced access to key goods and services (13).

It is also important to understand how adherence or resistance to COVID-19 containment measures affect community participation for people with disabilities trying to avoid contagion. The pandemic was particularly politicized in the United States, in which many conservative leaders dismissed COVID-19 as a hoax or championed individual rights over public health (14). This resistance to public health measures has been widely criticized for neglecting the welfare of PWD, effectively treating them as expendable or second-class citizens (15). Despite ample expert commentary, policy review, and journalistic coverage highlighting this tension, systematic research examining the everyday impact of COVID-19 noncompliance on people with disabilities remains limited. In an interview study, Lapierre and colleagues (16) found that older adults and individuals with disability experienced heightened anxiety, increased social isolation, and strained relationships with friends and family due to resistance to COVID-19 protocols. However, in response, this experience also fostered a sense of community and activism among individuals with disabilities (17).

PWD may face additional long-standing barriers to community participation and social engagement, such as lack of public accessibility (4, 7, 11, 18, 19). Still, little research has examined accessibility in the pandemic context. Notably, literature details issues with public transportation for PWD during the pandemic. Although a long-standing problem [e.g., (20)], transportation barriers increased during this pandemic, affecting PWD’s ability to complete essential tasks (13, 21). These barriers were particularly acute during the early stages of COVID-19, when transportation systems were adjusting and communication about service availability and protocols was lacking (21). Transportation barriers hindered access to essential goods and made employment difficult (10, 13).

There is also growing literature on the role of technology in enabling access to essential services during the pandemic for PWD. Telemedicine helped replace in-person visits, but ineffective technology could still cause delays in healthcare (22). Koon et al. (13) found that technology enabled community participation and socialization, but poor internet access was a problem for some. Additionally, Cho and Kim (23) found that while technology was crucial for individuals with disabilities, their usage rates remained the same as before the pandemic.

The pandemic also disrupted the personal assistance services (PAS) that many PWD rely on, which typically cannot be replaced by telehealth. Preexisting PAS workforce shortages worsened during the pandemic, driven by low wages and pandemic-related risks (24–27). However, during the pandemic, PWD may have needed more help from personal attendants to facilitate safety practices and social distancing (28). Difficulty finding and keeping reliable workers led to unmet care needs, negatively affecting both physical and mental health (5, 25, 29, 30). Additionally, PAS workforce shortages made it difficult for PWD to enforce their preferred COVID-19 safety practices when having to choose between risky care or no care at all (5).

Building upon the understanding of how COVID-19 has uniquely impacted PWD, particularly in terms of accessibility, healthcare, and personal care services, this study aimed to delve deeper into the lived experiences of people with mobility disabilities residing in community settings in the United States. As exploratory qualitative research, this study was guided by the broad research question: How did COVID-19 policies affect people with mobility disabilities in their everyday lives? The study aimed to capture the nuanced ways in which the pandemic, and the response to it, reshaped the lives of PWD, thereby contributing to a more informed and inclusive approach to public health policymaking in the future.

## Methods

This research was a sub-study within the Research and Training Center on Promoting Interventions for Community Living (RTC/PCIL) project. Aimed at increasing community participation among people with mobility disabilities, it was implemented in partnership with Centers for Independent Living (CILs). The emergence of the COVID-19 pandemic during the RTC/PICL’s implementation provided an opportunity to explore the lived experience of the pandemic on people with mobility disabilities. This was achieved by incorporating pandemic-related questions into the qualitative interviews of the program’s evaluation design. The research was guided by the social model of disability, which emphasizes societal barriers rather than individual impairments as the primary limitations faced by PWD. The study aimed to identify policies and resources supporting PWD’s community participation and health during the pandemic.

### Data collection and measures

The research team developed a semi-structured interview guide, which allowed for clarification and exploration of emergent ideas, to explore the effects of COVID-19 policies on people with mobility disabilities. This qualitative approach also facilitated understanding these policies within participants’ social, cultural, and environmental contexts. Interviews focused on the pandemic’s influence on participants’ daily lives (e.g., healthcare, assistance/supports, employment/education, finances, technology, groceries/prescriptions/errands), their lived experience with health and safety mandates, and their views on what policymakers should know about the disability community’s needs during COVID-19. The research team developed the initial interview questions based on early literature and media reports, and in consultation with the CILs. Our own research conducted early on in the pandemic (April–June 2020) also helped to inform this work. The questions were then refined through team-based discussion and in response to feedback from early interviews. A copy of the final interview guide can be found in the Supplementary materials.

Telephone interviews were conducted by two researchers and a graduate research assistant, all experienced in working with PWD. Notably, some members of the research team themselves had disabilities, adding valuable perspectives to the study. Despite their expertise and personal experiences, they did not have prior relationships with the participants, ensuring objectivity in the research process. Accommodations were provided during interviews, including options for written responses. Interviews lasted 45 min to an hour and were audio-recorded and transcribed verbatim. Participants received a $25 or $50 gift card, depending on the interview length. All procedures were approved by the Institutional Review Board at the University of Kansas.

### Study sample

Four CILs across the U.S. (in Georgia, Indiana, Ohio, and Pennsylvania) recruited 95 individuals who completed all stages of the RTC/PCIL, which was implemented from October 2019 to June 2022. Eligible participants were at least 18 years old, lived in the community, had a mobility disability/physical impairment, and were their own legal guardians. Everyone who completed the RTC/PCIL program post-survey were invited for a qualitative interview that included COVID-19 related questions. This process resulted in a sample of 76 participants. There were a few program participants (*n* = 19) who did not participate in the interviews due to either declining participation or could not be reached. For a detailed breakdown of participant demographics in this study, please refer to Table 1.

**Table 1:** Demographic characteristics of study participants (*n* = 76).

Characteristics	*n* (%)
Age	
18–34	10 (13.2)
35–64	53 (69.7)
65+	13 (17.1)
Gender	
Men	25 (32.9)
Women	51 (67.1)
Race	
American Indian/Alaskan Native	2 (2.6)
Black/African American	26 (34.2)
White	47 (61.8)
Other	3 (3.9)
Ethnicity	
Hispanic/Latino	1 (1.3)
Region	
Urban	72 (94.7)
Rural	4 (5.3)
Georgia	15 (19.7)
Indiana	13 (17.1)
Ohio	30 (39.5)
Pennsylvania	18 (23.7)

### Data analysis

The research team conducted a combined inductive and deductive thematic analysis (31, 32). Data immersion included reading and rereading interview transcripts to ensure familiarity with content and the broader context of participants’ lived experience. MAXQDA software was used to code and analyze the qualitative transcripts. The focus was on segments about the effects of COVID-19 on participants’ daily lives. Segmenting, a process of breaking transcripts into units of analysis, facilitated interrater reliability (IRR) testing. Two researchers conducted thematic analysis, drawing on the literature for deductive analysis and also inductively identifying emergent subthemes. They reached a consensus on the coding scheme before coding the transcripts. Interrater reliability was assessed among both coders for 19 transcripts, achieving a high level of agreement and reliability was reached (*r* = 0.90). Regular discussions were held to resolve any coding ambiguities, and the larger research team was consulted for consensus on discrepancies. Following the establishment of IRR, the remaining transcripts were independently coded by the two team members. Any coding difficulties were collaboratively resolved.

The analysis presented here originated by examining responses to the interview question “What would you want local and/or national policy makers to know about the needs of the disability community, especially during this time?” and categorizing the most common responses into three broad themes with subthemes. All interview data related to those themes was then integrated into these themes, including in responses given to other interview questions. The transcripts and coded segments were revisited multiple times to compare data with emerging themes and ensure that our interpretations were supported by the data.

## Results

Participants’ responses to the question about policymakers’ awareness of disability community needs during COVID-19 fell into categories of community participation, resources and support, and disability awareness. Over half the responses focused on issues unrelated to the pandemic, highlighting persistent struggles with accessibility, financial hardships, and lack of disability awareness. However, positives like increased telecommunication use and additional pandemic resources were noted.

### Community participation

The disability rights movement has always championed full community inclusion. However, during the pandemic, this ideal faced new challenges. Public health guidelines, combined with pre-existing issues related to accessibility, made community involvement more difficult for PWD. On a positive note, the increased use of technology during this time provided an alternative means of community participation and socialization for PWD, offering some benefits amidst these challenges.

#### Americans with Disabilities Act (ADA) accessibility

A notable portion of respondents emphasized the importance of improving ADA compliance within their communities. As noted by a female participant living in a Georgia metropolitan area, accessibility in their community is lacking despite the ADA being passed decades ago (ID 59):

*The ADA was passed in [1990], okay. There are several businesses that are not handicap accessible. For someone such as myself, that’s in a chair… policymakers or whomever need to really get on board with these things because we, meaning the disability community, we are human. We want to go places and travel and live our life. We spend money. We go places. We do everything. And I just feel like 2022 is not where it needs to be.*

Common issues included inaccessible building entries, bathrooms, and sidewalks. A few respondents noted that bathrooms that purport to be ADA accessible are not truly wheelchair accessible, as noted by a male participant living in a metropolitan area in Ohio (ID 26):

*Everybody basically says that they are handicap accessible. However, there’s a difference between being handicap accessible and being wheelchair accessible. The difference in the overall size and the spaciousness of a stall… and being able to maneuver in the stall and get around closer to the toilet.*

These challenges were more pronounced during the pandemic, affecting compliance with safety guidelines. For example, a few wheelchair users noted the difficulty of washing their hands frequently because of inaccessible sinks, as shared by a female participant with paralysis (ID 2):

*A lot of public bathrooms, or even at work… are not really accessible. So, I cannot pull up straight under them. I have to kinda turn to the side, which is hard with my back pain. So, I’m sore by the end of the day, because I’ve washed my hands so much, from having to turn sideways and bend over and contort into the sink.*

Another female respondent with a spinal cord injury noted that they rely on others to assist with heavy doors, but that this is more troublesome during a pandemic (ID 15):

*The doors give me a heck of a lot of trouble, so there’s times that I have to ask strangers to help me… I feel uncomfortable, though, when they are not wearing their mask.*

The poor state of sidewalks also posed additional hardships during the pandemic. As a result, it was difficult for PWD to walk/wheel places when these walkways were not navigable. Although spending time outdoors was advised by public health officials as one of the safest ways for people to get out of their homes and socialize safely during the pandemic, inaccessible sidewalks prohibited these activities, as noted by a female participant living in a Georgia metropolitan area (ID 50):

*A lot of the concrete is broken up, and [we] need accessibility, too. [We] have a right to… get a breath of fresh air.*

Some participants also noted that social distancing mandates and workforce shortages made it more difficult to access needed supports, including accessible public transportation. A couple participants with vision impairments noted that personal shoppers were no longer available, even after trying to work with stores to arrange this service in advance. A female participant with both mobility- and vision-related disabilities shared (ID 47):

*They were not offering personal shoppers, or any of that. It had to get to the point… I had to, in a very assertive way, say, ‘Hey, listen… You’re violating my ADA rights. So, you guys are gonna have to figure it out. And if you allow me to tell you what we could do, then we could make this work.’*

#### COVID-19 mandates

Respondents had varied experiences with COVID-19 mandates. While most understood their importance, difficulties arose, especially for those with underlying health conditions. Most respondents did not find it too difficult to follow mandates, beyond adjusting to new rules or the discomfort of masks. However, several others faced more serious difficulties with safety guidelines.

Several respondents noted ways in which it was difficult for them to comply with mandates due to inaccessible environments or the behavior of others. As shared above, inaccessible bathrooms made it difficult for wheelchair users to wash their hands. Individuals with vision impairments shared that it was difficult to tell if they were maintaining 6-foot distancing, and further, they could not see cues such as markings on the floor. However, one participant shared the usefulness of a smartphone app that enabled individuals with vision impairments to know how far they were from people. A few wheelchair users noted the difficulty in maintaining 6-foot distancing because able-bodied people would crowd into their space or cut in front of them in line when they were trying to maintain distance, as shared by one female participant (ID 2):

*For social distancing, I mean, it’s really dependent on other people. I cannot exactly scurry out of the way. I have to kinda operate my chair around other people. I feel like I do a pretty good job about it as far as people who are ahead of me. I really have no control of people who are behind me other than going forward and hoping they do not continue walking closely.*

Masks were cited as difficult to wear by some, with participants noting breathing difficulty related to asthma, PTSD, or other health conditions. Others noted the adverse impact of masks on effective communications. As shared by a female participant with both a mobility disability and asthma (ID 8):

*Wearing masks, and the distance, and the shields, and I’m hearing impaired, and I cannot read anybody’s lips with masks… It makes it a very big challenge to communicate or attend meetings. I need the distance for my health, but I also need to not have the distance for my health. So, it’s a very complicated situation… and then I’m asthmatic on top of it.*

Interestingly, most still felt it was important to wear masks despite these struggles since preventing the spread of COVID-19 was the more important priority, as demonstrated by a female participant with cerebral palsy (ID 60):

*It is difficult for me to put a mask on, but I’m glad to follow all mitigating practices…. I am more isolated and have dealt with that aspect well, but I will be ready to resume my activities in person when the virus is under control… I am handling that with the thinking I am saving my life and perhaps others’ [lives].*

Some coped with the difficulty of masks by taking frequent breaks from their masks when not near other people. Others limited their community outings, relied more on others, or relied on remote technologies for errands and appointments. For example, a male participant living alone decided (ID 6):

*It’s a lot easier to be in my house without a mask than to go into [the] community with a mask.*

Another strategy participants adopted was finding masks better suited to their needs. However, different mask styles were not always accepted by employers or medical providers, as shared by a male participant who found that a vented mask worked better for them (ID 22):

*They [medical providers] gave me a paper mask because I’m not allowed to have that [vented] mask, right? And I could feel the air coming in and out from around my eyes and the cheeks, and I’m like, I do not understand how this is more secure than the one I wear…. So, I do not see why I get such a problem with what I have.*

Additionally, some respondents did not find masks that truly met their needs as shared by one respondent who recommended the development of masks optimized for asthma.

Although some participants expressed these difficulties with wearing masks, participants tended to be far more concerned about resistance to COVID-19 safety measures by members of the public, including the anti-mask trend, as shared by a female participant with cerebral palsy (ID 16):

*But there are people who refuse to wear masks. So, that can be kind of discouraging, and it can affect the feeling of safety when I’m out and about.*

This situation contributed to increased anxiety and fears about catching COVID-19, especially among those who had health conditions which made them more vulnerable to the virus. As shared by a female participant using a Continuous Positive Airway Pressure (CPAP) breathing machine (ID 13):

*I did not have anxiety until the pandemic came…. It’s a lot of angry people that do not follow or just are not considerate of their health or no one else’s [health]… I think the anxiety part of it, too, where I cannot control what they do… But it just makes matters worse, or pisses me off more or less, when other people aren’t following the same guidelines, and they can be more detrimental to everybody’s health.*

There was also a sense of frustration observed regarding the misuse of ADA exemptions by some individuals not wearing masks. In contrast, it was noted that many in the disability community showed commitment to mutual health and safety, as previously mentioned, even when wearing masks is difficult. As shared by one female participant (ID 15):

*I see people with [cerebral palsy] wearing two masks to protect themselves from these assholes who go into Walmart and will not wear their masks properly or at all… ‘Oh, I have a breathing problem.’ But you are able to yell. You’re able to do this, that, and the other.*

Some participants remained home more when their disability or health conditions made it difficult to wear masks, however, as an emergent theme, we found it was far more common for participants to report avoiding community outings due to mask resistance by community members. As stated by a male participant (ID 72):

*I try to stay away from as many people as I can because I do not know what other people are doing. And some people, they do not wear masks… Nah, I’m not bein’ in that environment. I go back home. I stay at home more.*

A female participant switched to online shopping in response to poor community adherence to mask mandates, but shared (ID 15):

*I want to be able to go out and do some Christmas shopping, but I wanna be able to do so safely. And sometimes I need something that I cannot get through a pickup or delivery.*

Conversely, when others complied with mandates and safety practices, this expanded opportunities for community participation and socialization. Participants felt safe returning to work or engaging in social activities when they were confident that colleagues, family, and friends were taking COVID-19 precautions seriously. As shared by one female participant (ID 2):

*I do go into the office just for productivity reasons. But I have my own office. We wear masks when we are in the hallway. We social distance, so there’s all the PPE, hand sanitizer, and all that. So, I feel safe.*

This compliance by others created safe bubbles where people could get out of their homes or engage with others, which was important for reducing social isolation and improving mental health.

For these reasons, improving compliance with COVID-19 safety mandates was a commonly cited policy recommendation, as shared by a female participant with cerebral palsy (ID 16):

*People do not really realize that the safety of people with disabilities is sometimes even more at risk than the general population… It’s become very difficult, rather quickly, to kind of live life as normal when you are stuck inside all the time to try to keep yourself and those around you safe and healthy. So, I think I would like policymakers to kind of know and have an understanding…. I feel as though some policymakers, although, of course, not all, took health and safety guidelines more seriously than others. So, I think for those who maybe did not take it as seriously, I would just encourage them to think about why those guidelines and policies to ensure everybody’s health and safety are so important… not only to everyone, but especially those in the disability community.*

#### Technology-facilitated participation

In contrast to the barriers to community participation posed by inaccessible environments and public resistance to COVID-19 safety guidelines, the expanded use of technology during COVID-19 offered many benefits and was commonly cited as a positive outcome out of the pandemic. Some respondents noted that these technologies were long available, but increased use during the pandemic expanded opportunities for community engagement or more convenient ways to meet essential needs. The increased availability of online ordering for groceries and other supplies, either for delivery or curbside pickup, not only helped PWD disabilities practice social distancing, especially when community adherence to safety guidelines was low, but was also beneficial in reducing reliance on others. As a male participant stated (ID 6):

*Technology, using a smartphone to order stuff and pick it up at the store, I think pushed even faster with COVID … [which] is a major thing for a person to be more independent instead of depending on other people.*

Similarly, another participant, female, expressed preference for curbside grocery pickup due to its time-saving convenience and the added benefit of avoiding difficulties like navigating snow in the winter (ID 76):

*I actually am still doing curbside pickup with grocery shopping. Only because, selfishly on my part, it saves me so much time … So, I’m not sure if I’ll ever go grocery shopping again if they keep the curbside pickup available…. it just makes things much easier for me. Especially in the wintertime because then I [do not] have to worry about getting out of my vehicle and going through the snow.*

Respondents were also generally favorable about the shift to telehealth, noting the convenience in accessing healthcare. Telehealth eased transportation pressures, even as shared by a female participant who reported having reliable transportation (ID 41):

*[Telehealth] was new after COVID. But I like it a lot … I’m not having to schedule transportation and everything to get back and forth to* var*ious appointments like I have.”*

It also saved time and reduced fatigue or pain, as noted by another participant (ID 52, female):

*The best thing, to me, is that most of my doctors are virtual, and I do not have to get dressed and get out of my house. Yeah, especially with my pains and aches. I love that. I really do.*

The pandemic also expanded access to technologies that enabled remote health monitoring, as shared by a male participant who was able to get a smart watch (ID 15):

*They’re working on adding a blood pressure [to my smart watch], but it constantly monitors my tachycardia … It also does ECGs and has fall detection…. So, I’d have to say getting that watch and learning to use it would not have happened without COVID. -.*

Some participants also noted the benefit of remote work or school as a PWD, which provided more flexibility to accommodate disability-related needs and eased transportation burdens. As one participant noted (ID 36, male):

*Because of the pandemic, a lot of the classes that I’m taking are virtual classes. It saves me gas money … It does make my life more convenient, and it also helps the lives of my caretakers because most of my classes are online now. So they can just come over kind of whenever they want to take care of what I need them to take care of because my schedule’s a little bit more lenient and flexible.*

Additionally, a male participant noted that the widespread shift to teleworking created a more equal environment for PWD (ID 61):

*[Working remotely] wasn’t new to me. The hybrid work model, back in the day, was taboo. ‘People who worked from home are just looking for excuses not to work’ was kind of the thought process. And I think, for me, I see a lot of businesses saying it’s more acceptable. That they realize that people aren’t lazy, they are not sitting at home and not working…in some ways, they are more efficient in homes … If it wasn’t for COVID, and if my doctor said I can only go in the office one day a week, I truly believe I would be laid off. I think COVID changed the mindset of how a work environment can function.*

Participants noted that recreational and social activities were also expanded due to increased use of social media or teleconferencing. A female participant described how she became more involved with her congregation during the pandemic (ID 27):

*We meet more often now than what we did before the pandemic because we do everything online, all of our meetings… And witnesses are known to go door to door. Well, we are not going to do that… So, we do the letter writing… together on Zoom.*

Additionally, several participants noted increased engagement with friends and family as their schedules became more flexible and they adopted new means of communication. As a male participant stated (ID 61):

*We had four generations join a Skype video call… We could have done this with ease, anytime we wanted [before the pandemic]… We never used it. The pandemic provoked [using Skype]. There’s like 10 of us, and some of us from multiple states… So it forced us to use the technology to actually see each other.*

However, there were also barriers to accessing technology. For example, some older participants reported not being comfortable using these technologies or navigating a steep learning curve, as shared by one participant in her late 50’s (ID 35):

*Just trying to download a file and then find it again. I get mixed up with that. And then I get frustrated… But for the most part, technology has been very helpful.*

Others struggled with knowing how to use accessibility features in technology, as shared by a female participant with hearing impairments (ID 8):

*I have learned to use a lotta stuff that’s on Zoom and online, but I still have to have someone with me because my hearing aids do not cooperate with Wi-Fi signals and technology. And so, when I’m on a Zoom, it’s screeching and stuff in my ear… I think it would be awesome if there was some Zoom tutorials on, ‘Hey, if you are hearing impaired, you can have this option and you just click this button.’*

Reliable, affordable internet was also a challenge as shared by one participant (ID 59, female):

*I do not have Wi-Fi. I cannot afford it… I have to buy groceries. I have other stuff to do with my money.*

One participant shared concerns in trying to operate an online business due to dropped Zoom calls. For these reasons, a few participants advised that policymakers improve access to internet and technology, as demonstrated by the following participant (ID 54, male):

*Internet is vital for people, especially when you are looking at doing telemedicine, so that you do not have to risk yourself trying to find a ride to get to a doctor and risk yourself going into that environment… So yeah, transportation [and] internet, are both very, very important things to have in any kind of community.*

### Resources and support

In their recommendations to policy makers, participants widely pointed to the need for more resources, including transportation, basic living expenses, and personal assistance services. The COVID-19 pandemic posed both constraints and barriers to accessing these resources.

#### Transportation

Many participants wanted policymakers to improve access to affordable, reliable transportation for PWD, a theme that also intersects with community participation, above. Many participants do not drive or own a personal vehicle and thus depend on public transportation, which is key to their independence. As noted by a female participant (ID 16):

*I do not drive, so either I use the paratransit service, or I ask friends or family to help get me to where I need to go… Usually, I’m able to get out into my community independently as long as I have some form of transportation.*

The pandemic had a major impact on the access to safe, reliable transportation. One participant noted the advantage of having bus fare waived in their metro area during the pandemic (ID 41, female):

*[The fare was] typically I think $3 a trip, [but now] it’s free… and all the buses are handicap accessible.*

More often, however, participants spoke of pandemic-induced barriers. Many participants were not comfortable using public transportation during the pandemic due to concerns about increased exposure to the virus and low community adherence to safety measures, as shared by a female participant (ID 37):

*I was a little worried [using] medical transport… they were not masked in the vehicle. It concerned me because you do not know who that other person’s been around.*

This concern also applied to rideshare services as participants felt they cannot know if the drivers are really taking the necessary safety precautions.

Further, others noted how new paratransit rules that limited the number of riders, intended to promote COVID-19 safety, combined with a shortage of drivers, made public transportation less reliable. Participants reported increased difficulties in scheduling paratransit, even with advance notice. They faced longer waiting times and stricter constraints on their return schedules. A few participants reported instances of being left waiting outside for extended periods because paratransit services were restricted to transporting one person at a time, as experienced by a female participant (ID 47):

*When the bus pulled up, I expected to get on, and he said, ‘Well, I’ve already got a person on, so you are gonna have to wait another hour for another paratransit.’ And, I mean, it was a hot day, and I was just ready to get on the bus and go home. And it was just like, well, what can I do? There’s nothing to do.*

Participants living in suburbs or rural areas without public transportation highlighted the prohibitive costs of using services like Uber and Lyft. As noted by a female participant (ID 35):

*And where I live… a suburb of [a large city]… there’s no buses that come out here… So, I was using Lyft or Uber. And that got to be expensive.*

Further, taxis and rideshare were often not accessible for wheelchair users. Finally, some participants noted the increased cost of transportation due to the rising cost of fuel. Although transportation is an essential resource for remaining independent and participating in the community, participants noted the myriad ways that it was impacted by the pandemic.

#### Basic living expenses

In their recommendation for policymakers, over a quarter of participants pointed to a need for more resources or programs to help cover the basic costs of living, which in addition to transportation, described above, included food, housing, and durable medical equipment. The difficulty covering disability related expenses or surviving on a fixed income was a long-standing concern, pre-existing the pandemic, as noted by a male participant living alone (ID 46):

*When it comes to housing, how can you expect an individual to [afford] that if [they] only get what, $800 or $900 a month through SSI? Or, let us say, $1,000 with SSDI? And you are saying, ‘Okay, for you to live here, you have to be making two or three times the rent.’ That’s not gonna happen.*

On one hand, participants noted that the additional financial resources distributed during the pandemic, such as the stimulus checks and increased Supplemental Nutrition Assistance Program (SNAP, commonly known as food stamps), were beneficial. As noted by a female participant (ID 41):

*Honestly, the increase in food stamps… that made a world of difference.*

Additionally, revised policies that allowed SNAP users to order groceries online for pickup were also seen as helpful. However, on the other hand, the increased cost of living made it more difficult for some people to stretch their dollars, which limited the impact of the financial stimulus. As noted by a female participant (ID 45):

*To be honest with you, I appreciate my extra food stamps… but I’ve still not really been able to get everything I need because the prices of everything [increased].*

Additionally, several participants found grocery delivery fees difficult to manage, and SNAP users noted that delivery was not always allowed despite the benefits of grocery delivery for individuals with mobility disabilities, transportation barriers, or at increased risk of contracting COVID-19. A female participant advised that policymakers address delivery fees for people with disabilities, sharing (ID 40):

*A lot of these different services for getting essentials, they cost extra money… I might not have the extra $10 or $20 to pay in fees. So, I’ll go to the store, and I might be in pain, or I might be stiff, or I might be nervous about the Corona [virus].*

Some participants also shared that it was difficult to find resources to help cover essential living expenses or that the criteria for these programs were too restrictive. For example, a couple participants noted they did not meet the age criteria for assistance programs. Another participant shared that income limits for his state’s Medicaid Buy-In program, essential for covering his disability-related healthcare costs, had adverse consequences on his career and wages (ID 75):

*Without my [Medicaid], I pretty much could not work. However, with [Medicaid Buy-In program], they make it so you can only have so much money in the bank. I’ve had to turn down two pay raises because it would put me over my limit.*

Finally, some participants noted that merely achieving a basic standard of living is not sufficient for truly thriving in their communities, as demonstrated by a female participant (ID 37):

*[Policymakers] have no clue what it’s like to be dirt poor… I do not know what I tell them other than you have no idea how hard it is… If you do not have money, you do not go anywhere. You do not do anything. You just, you are home. And that’s it… You cannot afford to do social things anyways.*

#### Personal assistance services

Personal assistance (PA) services are essential for keeping many PWD out of nursing homes. In some ways, this support became even more important during the pandemic. For example, one participant spoke of needing to secure additional PAs to ensure coverage when other PAs were out due to COVID illness and quarantine. Several participants reported relying more on PAs to run essential errands so that they could avoid crowds, as noted by a female participant (ID 60):

*[During the pandemic] I use my attendants to complete tasks that normally I would do*.

However, there was some uncertainty among participants about whether this workforce was considered essential and thus should continue providing support during the pandemic. Consequently, they wanted to emphasize to policymakers the necessity of their service, as suggested by a female participant (ID 23):

*Home Health Care still needs to happen. We do not have a choice as to whether or not we need [them]. Our personal needs need to be cared for like going to the bathroom or taking a shower.*

Additionally, many participants faced challenges in finding and retaining competent PAs, a problem exacerbated during the pandemic. They advised policymakers to address this issue, which is rooted in low wages, and has worsened recently, as highlighted by a female participant (ID 28):

*Home care has really gone down the drain… To get some decent help is really hard. [Personal assistants] just do not want to do nothing for nothing, you know? They do not get paid, right?... That’s why it’s always been like that, but it’s gotten really bad.*

Participants advised that policymakers improve wages for this workforce, as well as expand programs that allow PWD to access this service because it is also too expensive to pay for out-of-pocket services on a limited income.

Finally, while some participants commended their PAs for taking extra precautions during the pandemic to enable safe care, others expressed difficulty finding workers who would comply with health mandates or were properly trained on infection control. The female participant, quoted immediately above, expanded (ID 28):

*I just think they need to hire more people [and] make them have the vaccine and wear a face mask, but some refuse… If you do not, then you should not have your job... I just do not like being around those that are not vaccinated. I’m just not comfortable with that. I have medical issues.*

Thus, the politicization of COVID-19 and resistance to safety protocols not only limited community participation, as demonstrated above, but also made it more difficult to find PAs who participants felt were safe to bring into one’s own home for this essential service.

### Disability awareness and visibility

Over a fifth of respondents emphasized the need for greater disability awareness and visibility. They often felt overlooked or treated as an afterthought in policymaking. Additionally, there was concern about being perceived as a homogenous group when addressing disability issues. Participants suggested ensuring representation in policy development and implementation by including specific roles or “seats at the table” for relevant stakeholders. Participants emphasized the importance of increasing disability awareness and visibility to combat the dehumanization and overgeneralization often seen in policymaking. Many participants felt that policymakers were indifferent or dismissive toward people with disabilities, a sentiment echoed by a female participant (ID 16):

*I just do not like to be cast out in the cold [like] dogs. They treat their pets better than they treat the disabled people. Or the older adult. You know, it’s really sad.*

Some participants were uncertain what they wanted policymakers to know because they felt leaders were not listening and did not care about them. As stated by a male participant (ID 17):

*At the end of the day, the only reason why people would stay silent about their problems is because they feel like there’s no one that genuinely… gives a damn about them.*

As noted by a male participant, this dehumanization was exemplified when former President Trump mocked people with disabilities during his campaign (ID 75):

*I always would like things to be a little better. I do not want Presidents making fun of people’s disabilities on national TV.*

As shared by another participant, this dehumanization was also seen in care rationing policies that threatened access to treatment for PWD (ID 16, female):

*For those in the disability community who ended up being hospitalized for COVID-19, I think care rationing was a very real and very difficult concern and something that’s definitely happened… Perceived quality of life should not be taken into consideration when you are choosing whether or not to save someone or to cure them of their illness.*

As suggested by one participant, policymakers do not fully understand the diverse needs of the disability community. Instead, they often resort to a one-size-fits-all approach in policy formulation, based on inaccurate perceptions of what these needs entail (ID 41, female):

*As a community, we are not a monolith. We do not all need the same thing. And just because whatever this may be may work for Billy, it does not mean it’s gonna work for Bob. Get more individualized solutions in place.*

To address these concerns and ensure that policies consider the unique needs of the disability community, participants widely pointed to the need for improved disability awareness and visibility. Participants stated how important it is that their community is seen and heard, in a global pandemic and beyond, in regard to what policymakers should know. As shared by a male participant (ID 56):

*It is absolutely imperative that [policymakers] …encourage them to come to the table and be a part of the decision-making process and not make any assumptions based on some stereotype as to what will be effective for them.*

In addition to a seat at the table, participants spoke to a general need for improved awareness of the disability community and their needs, as expressed by a female participant (ID 33):

*More inclusivity [is needed] in order to understand people like us. And sometimes people do not want to understand that people are disabled… People need to be more patient, understanding, and stop being arrogant… Then people want to say, ‘Hey, this person is just like me but a little different, you know?’ [Disability issues] just need to be advertised, more awareness. Billboards, posters, videos, whatever.*

## Discussion

Policy responses to the pandemic had a complex and mixed influence on PWD, simultaneously enhancing and constraining their quality of life. Initially, it was evident that pre-existing deficiencies in infrastructure and support were exacerbated during the public health emergency. This situation made it more challenging for PWD to cope with the pandemic, comply with key COVID-19 safety protocols, and meet their basic needs.

Inaccessible environments hindered PWD from adhering to public health guidelines, affecting activities from handwashing to utilizing outdoor spaces safely. Transportation was another significant challenge, echoing previous research findings (13, 21). Public transport was perceived as risky, private on-demand services were unaffordable, and public on-demand services suffered from limited availability and long wait times due to workforce shortages. While economic assistance, such as expanded SNAP benefits and stimulus checks, proved beneficial for many during the pandemic, others struggled with the escalating cost of goods and living. These costs were particularly problematic for those dependent on disability income or constrained by Medicaid Buy-In and other Medicaid asset rules. Moreover, many PWD rely on low-wage PAs, who became harder to hire and retain during the pandemic, which also reflects previous research findings (5, 24–26). Participants in our study also noted an increased need for assistance from others, including PCAs, to help reduce exposure to COVID-19.

### Practice and policy implications

Now that the COVID-19 public health emergency is officially over, it is crucial to reflect on which policies were effective and which were not, to prepare for future public health emergencies. This reflection should also consider how beneficial policies and practices can support ADA compliance, compliance with public health measures, community participation, and overall quality of life.

The pandemic highlighted the potential of technology to enhance community living for PWD, including convenient healthcare access, new opportunities for remote work and volunteerism, and improved social engagement. The pandemic accelerated the use and development of these technologies across various sectors, while at the same time revealed shortcomings of these technologies. As life returns to “normal,” it is important that the use of remote technologies continues as a new standard and are accessible to all. Equitable access includes affordability, skills development, and knowledge accessibility features.

Our study showed that remote work is not only feasible but often beneficial for PWD, helping them reduce virus exposure and manage their health and disability needs. As the debate around remote work continues, it is vital that this option remains available for workers with disabilities as a reasonable accommodation. Indeed, the U.S. Equal Employment Opportunity Commission (EEOC) identifies telework as a reasonable accommodation when essential job duties can be performed remotely (33). However, prior to the pandemic, employers won most rulings on whether they could reject an employee’s request for remote work, often citing the need for teamwork, supervision, or security concerns. Yet, remote work during COVID advanced remote work technologies and work culture (34). In their updated guidance for the post-COVID period, the EEOC notes that telework during COVID-19 can serve as a trial period in considering whether a PWD can continue to meet job expectations through remote work (35). However, offering remote work exclusively to PWD may foster stigma, a concern expressed by a study participant, highlighting the need for better disability awareness. Additionally, workers with disabilities are underrepresented in professional and white-collar occupations with high potential for home-based work, thus indicating a need for improved opportunities in these job sectors (36).

Many participants also showed a preference for telehealth and online shopping, indicating a desire for these services to continue. Telehealth can lower the cost of care, reduce transportation costs, and decrease the need for personal care attendants (37), however, infrastructure and access barriers persist, from reliable internet and equipment access to the need for user-friendly interfaces (22). Ensuring telehealth is accessible and covered by insurance, including Medicaid, is important. Ongoing funding for technologies supporting telehealth, like health monitors (e.g., heart rate or blood pressure monitors), is also necessary. Moreover, policies that allowed SNAP benefits for grocery delivery or curbside pick-up should be continued and expanded to include delivery costs. Equitable access to remote technologies is crucial to sustaining these benefits for PWD. Additionally, while grocery delivery expanded food access by overcoming transportation barriers for many, this remains a challenge in many rural areas where food delivery is not available (38, 39), and barriers to grocers being able to offer food delivery in rural areas also need to be addressed.

Although the United States federal government committed $1.8 trillion in economic stimulus funding to individuals and families during the pandemic, which included benefits such as expanded food assistance, expanded unemployment income, and stimulus checks (40), many participants still struggled with the cost of living, including food, housing, and transportation. This reflects a larger body of literature finding that the COVID-19 pandemic exacerbated existing financial disparity and targeted financial resources for PWD were insufficient in many countries (41). In future public health emergencies, funds should be more strategically targeted to individuals with lower, fixed incomes and proactively address the unique challenges that PWD face in accessing affordable housing, transportation, and other essential services.

The federal government also allocated an additional estimated $12.7 billion dollars to states for their Medicaid Home and Community Based Services (HCBS) program through the American Rescue Plan Act (ARPA) (42). The HCBS program provides long term services and supports, including personal assistance services (PAS), to low-income citizens in home and community-based settings. Yet, despite this funding surge, a large body of research echoes the concerns of our research participants that the PAS workforce shortage crisis worsened during the pandemic (5, 24–26). Additionally, this essential workforce was invisible during the pandemic with uneven access to key benefits, such as hazard pay, sick pay, or personal protective equipment, needed to help mitigate pandemic risks (5, 43). This persistent challenge despite increased funding may reflect that fact that most ARPA HCBS investments into the direct care workforce were temporary in nature, such as wage bonuses, rather than long term investments because ongoing funding requires a commitment from state legislators in state budgets. Among the four states of the participants in our study, only Pennsylvania used these funds to support permanent direct care rate increases (42). Our data does not support a robust comparison of participants across states on this issue, but comparative research of this nature is warranted to better understand the impact of different spending priorities on outcomes for the direct care workforce. Such research can help inform priorities and strategies for long term investments in direct care workforce wages, benefits, or skill development. Ultimately, a stronger direct care workforce can improve outcomes for care recipients, including giving PWD more choice in recruiting workers who best meet their care expectations. The new Medicaid Access Act is also a promising step toward strengthening this workforce by requiring that at least 80% of Medicaid payments for direct care go directly to worker compensation. This act will also improve data collection for this workforce (44), which will provide much needed data that researchers can use to further assess workforce gaps, needs, and outcomes.

Not all people with disabilities were at a higher risk of adverse COVID-19 outcomes, but many were, and their perspectives should have been central in COVID-19 policy decisions. This means not just implementing protective policies, but actively including PWD in the policy making process, thus echoing the call made by Campbell et al. (45) over a decade ago when noting that pandemic planning was ill equipped to meet the needs of the disability community, even though they face increased risk of contagion as well as service interruptions. Public health mandates were a mixed experience for PWD. Most tried to comply, even when challenging, and could benefit from more supportive environments, flexible policies, and tailored resources and funding. The misuse of ADA exemptions by anti-maskers posed significant risks to the disabled community and limited their community participation. The resistance to public health measures also affected the ability to find PAs willing to follow health guidelines. Additionally, participants noted increased risks from able-bodied individuals, such as those crowding wheelchair users. This highlights the importance of including disabled voices in public health policy creation and implementation and amplifying disability visibility.

### Strengths and limitations

This comprehensive study has several strengths and limitations. The qualitative interview design explored the influence of COVID-19 policies on people with mobility disabilities from their own perspectives, with the semi-structured interview design revealing important emergent themes and the issues that were of most concern to participants. This approach yielded a wide range of responses that highlighted diverse experiences with COVID-19 and the life contexts that shaped these experiences. However, the interviews reflected a single point of time and were not solely focused on COVID-19, which might have limited the depth and specificity of responses related to the pandemic and its consequences for PWD. Follow-up interviews would help collect thicker descriptions and a more nuanced understanding of how the pandemic affected participants over time. The telephone interview approach allowed participation across multiple states but limited our ability to capture non-verbal communication.

The sample, while diverse in terms of race, gender, age, socioeconomic status, and type of mobility-disability, was more limited in regard to some positionalities or standpoints. Although we interviewed participants across four states, the sample was overwhelmingly urban, which may not accurately reflect the experiences of PWD living in rural areas. The rural, disabled population is particularly underserved (6), and may have different attitudes toward or concerns about COVID-19 public health measures (46). The study also did not include individuals living in nursing homes or assisted living facilities at the time of the interviews. As the study did not include a specific focus on nursing home or assisted living residents, where COVID-19 protocols were more strictly enforced, there may be a lack of insight into how these individuals perceived COVID-19 mandates and their autonomy in decision-making. Thus, additional research is needed to better understand the experiences of other subgroups of people with disabilities, including those living in rural areas and in congregate settings.

## Conclusion

In conclusion, the pandemic’s policy response significantly shaped quality of life for people with disabilities (PWD), a newly recognized health disparity population, both positively and negatively. This research illustrates how disruptions in services and increased isolation have exacerbated mental health challenges among PWD, highlighting the urgent need for integrated health services and robust social support systems. These challenges underscore existing deficiencies in infrastructure and support systems, emphasizing the importance of economic assistance, yet also emphasizing the struggles faced by those reliant on disability income or limited by Medicaid rules. Moreover, the pandemic has highlighted critical health implications, as PWD faced heightened risks of adverse health outcomes due to disrupted services and increased exposure to the virus. The study emphasizes the crucial need for including PWD in policymaking, particularly for public health decisions, to address their unique needs, including policy measures that support the capacity of PWD to comply with public health measures as well as protecting their safety in public spaces. Additionally, the findings support the need for continuous evaluation and adaptation of policies to prevent further marginalization of disabled populations in the wake of global health crises. As society transitions post-pandemic, it becomes vital to maintain and enhance the use of technologies that have proven beneficial for PWD, like remote work, telehealth, and online services, ensuring equitable access and effective utilization. This study serves as a call to action for improved disability awareness, policy inclusion, and tailored solutions to better the lives of PWD in all societal aspects.

## Data availability statement

The raw data supporting the conclusions of this article will be made available by the authors, without undue reservation.

## Ethics statement

The studies involving humans were approved by University of Kansas Human Research Protection Program (STUDY00144244). The studies were conducted in accordance with the local legislation and institutional requirements. The ethics committee/institutional review board waived the requirement of written informed consent for participation from the participants or the participants’ legal guardians/next of kin because recorded oral informed consent was instead obtained instead to support remote interviews competed by phone or teleconference.

## Author contributions

CW: Formal analysis, Writing – original draft, Writing – review & editing. RG: Formal analysis, Investigation, Writing – original draft, Writing – review & editing. KG: Investigation, Project administration, Supervision, Writing – original draft, Writing – review & editing. JH: Funding acquisition, Investigation, Methodology, Supervision, Writing – review & editing.
