# Supplementary material for: Navigating new normals: the influence of COVID-19 policies on community access and well-being of people with mobility disabilities in everyday life
Source: Front Public Health. 2024 Jul 4;12:1401777. doi: 10.3389/fpubh.2024.1401777 (PMC11254613; doi:10.3389/fpubh.2024.1401777)
Supplement: Supplementary file 1 [file Table_1.DOCX]

**Supplemental Table 1: Qualitative Codes and Definitions**

| **Code** | **Definition** |
| --- | --- |
| Community Participation | When participants state/infer that they are going out into the community, participating in the community, or unable to go out in the community or participate in the community. Examples include education, employment, social outings, recreation, and errands. Include in-person or virtual participation. |
| Fear | When participants express fear or anxiety or related emotions around the impact of COVID for themselves or others. For example, participant is afraid of catching or spreading COVID, afraid that they will have very serious or long-term effects due to having COVID, concerned about vaccine safety, etc. |
| Financial | Participants mention having a financial/economic effect (negative or positive) due to COVID or COVID policy. |
| Healthcare access | Accessing health care during the pandemic, of any type (primary, specialty, mental health, therapies, prescriptions, etc.) (Note, do not include personal care attendants, include this under “reliance on others” code). |
| Health and Function | Any impact (positive or negative) on health and function of participant as a result of COVID or COVID policy; defined broadly to include physical health, mental health and wellbeing, and functional status. |
| Home | The participant’s home living environment, including who they live with and the physical home environment. |
| Policy | When participant refers to health or economic policies related to COVID- this could be local, state, national. Also includes discussion of adherence to COVID public health protocols, by self or others. |
| Positives | The benefits of being in a global pandemic. When ppts talk about what the positive or good things that came out of the pandemic. For example, the use of groceries being delivered more widely. |
| Pre-COVID | The participants baseline before the pandemic, in any area. |
| Reliance on others | Reliance on others during the pandemic, including family caregivers, friends, and Personal Care Attendants; increased independence or dependence on others during the pandemic. |
| Socialization | Connection the participant had with socializing or being social with others during the pandemic, such as family, friends, and neighbors. |
| Technology | When participants refer to using technology to maintain their existence or engage in activities during the pandemic. Example: socialization, telehealth, ordering groceries, remote work, distance learning, etc. |
