# Supplementary material for: Navigating new normals: the influence of COVID-19 policies on community access and well-being of people with mobility disabilities in everyday life
Source: Front Public Health. 2024 Jul 4;12:1401777. doi: 10.3389/fpubh.2024.1401777 (PMC11254613; doi:10.3389/fpubh.2024.1401777)
Supplement: Supplementary file 2 [file Table_2.DOCX]

**Supplemental Material: Semi-Structured Interview Guide (Final Version)**

*Thank you for participating in this interview. We will keep this brief, and just want to learn about your experience with the COVID-19 pandemic, and your experience with the research program you recently completed with [CIL Staff Name].*

**COVID-19 subset** (alternatively asked before or after the program evaluation questions):

*Let’s discuss the COVID-19 pandemic…*

1. Have you or someone you know been directly affected (e.g., tested positive, had a family member test positive) by COVID-19?
   - If yes, please elaborate
2. Do you have any difficulties with wearing masks, washing hands frequently, or keeping six feet away from people in public places?
   - Probe: Does this impact your ability or decision to go out in the community?
   - Probe, if a challenge is mentioned: What strategies have you tried to handle this challenge(s)?
3. What sorts of things have changed for you as a result of the COVID-19 pandemic?
   - Access to healthcare?
   - Access to assistance or supports (e.g., PCAs)?
   - Employment/education/financial?
   - Use of Technology?
   - Any other changes (e.g., physical abilities; access to groceries, medical supplies, Rx’s)?
   - Probe, if a challenge is mentioned: What strategies have you tried to handle this challenge(s)?
4. What would you want local and/or national policy makers to know about the needs of their disability community, especially during this time?
5. What is the best thing that’s come out of this pandemic for you?
